# Wild birds respond to flockmate loss by increasing their social network associations to others

**DOI:** 10.1098/rspb.2017.0299

**Published:** 2017-05-17

**Authors:** Josh A. Firth, Bernhard Voelkl, Ross A. Crates, Lucy M. Aplin, Dora Biro, Darren P. Croft, Ben C. Sheldon

**Affiliations:** 1 Edward Grey Institute, Department of Zoology, University of Oxford, Oxford OX1 3PS, UK; 2 Department of Zoology, University of Oxford, Oxford OX1 3PS, UK; 3 Animal Welfare Division, Vetsuisse Faculty, University Bern, Laenggassstrasse 120, 3012 Bern, CH, Switzerland; 4 Fenner School, Australian National University, Canberra 2601, Australia; 5 Centre for Research in Animal Behaviour, College of Life and Environmental Sciences, University of Exeter, Exeter EX4 4QG, UK

**Keywords:** social structure, social networks, population declines, social perturbation, social foraging, social bonds

## Abstract

Understanding the consequences of losing individuals from wild populations is a current and pressing issue, yet how such loss influences the social behaviour of the remaining animals is largely unexplored. Through combining the automated tracking of winter flocks of over 500 wild great tits (*Parus major*) with removal experiments, we assessed how individuals' social network positions responded to the loss of their social associates. We found that the extent of flockmate loss that individuals experienced correlated positively with subsequent increases in the number of their social associations, the average strength of their bonds and their overall connectedness within the social network (defined as summed edge weights). Increased social connectivity was not driven by general disturbance or changes in foraging behaviour, but by modifications to fine-scale social network connections in response to losing their associates. Therefore, the reduction in social connectedness expected by individual loss may be mitigated by increases in social associations between remaining individuals. Given that these findings demonstrate rapid adjustment of social network associations in response to the loss of previous social ties, future research should examine the generality of the compensatory adjustment of social relations in ways that maintain the structure of social organization.

## 1. Introduction

The loss of individuals from wild populations can have many consequences for the remaining animals. For example, the remaining individuals may benefit from increased survival or reproduction due to reduced competition [1–3]. Alternatively, individual fitness could be reduced if such loss increased predation risk or decreased the potential for beneficial interactions, such as mating opportunities or cooperation with others [4–6]. The immediate consequences of losing members of a population are likely to depend on how the remaining individuals interact with one another (i.e. the resulting social structure). However, the consequences of the loss of individuals for social structure remain largely unknown.

Recent developments in animal-tracking technologies and analytical methods now allow the fine-scale assessment of individuals' social associations to one another [7,8]. In this way, social structure can be quantified as a social network [9–12], and this approach has now been applied to a wide variety of wild animal societies [13]. Such networks are known to relate to various biological processes, such as transmission of disease and information [14–18], various measures of fitness [19–21] and how selection operates [22,23]. The individuals that are central in a network (i.e. with high social connectedness) differ from peripheral individuals both in terms of their extent of influence on the social system, as well as their social experience and the pressures exerted upon them [11].

Previous studies of vertebrate populations have demonstrated that individuals are repeatable and consistent across time in the social positions they hold within the network [20,24,25]. While an individual's network position may rely upon its own attributes and behaviour, it also intrinsically depends upon which other individuals it interacts with [26]. How changes in the composition of social groups influence individuals' social associations is therefore a fundamental question of group living. Nevertheless an empirical assessment of the direct consequences of one of the most significant of such changes—the loss of individuals—is currently lacking.

Research into general network theory has typically considered the effects of the loss of certain ‘nodes’ (individuals) by simulating node removal and assessing the resulting network structure [27,28]. Applying these simulation approaches to wild mammalian populations has shown that the removal of socially central individuals can fragment social networks or increase the social distance between individuals [29–32]. While such approaches are potentially very informative, their validity is currently limited due to the lack of knowledge regarding how the loss of individuals may affect the behaviour of the remaining members within natural populations [33–35].

Previous experiments have been mainly limited to captive, rather than wild, animal groups. These experiments have indicated actual individual loss may indeed have different consequences than those expected from simulations. For instance, within captive pigtailed macaques (*Macaca nemestrina*), the removal of high-ranking group members caused more extensive social dissolution than that predicted by simulations, as these individuals promote social cohesion [36]. By contrast, in captive Indian queenless ants (*Diacamma indicum*), the removal of central individuals had less impact than simulations predicted [37], as remaining individuals upregulated their activity [37]. Similarly, analyses tracing the changes in path length and connectivity in experimental colonies of social wasps (*Ropalidia marginata*) following removal of individuals demonstrated that the redundancy within the original network provided substantial resilience to losses [38].

In this study, we experimentally test the social consequences of the loss of individuals from a wild population of great tits (*Parus major*). Through tracking the flocking patterns of over 500 individuals using the large-scale deployment of radio-frequency identification (RFID) technology, we monitored individuals' positions within the social network while temporarily removing birds from the population. In this way, we directly assess how birds that lost their flockmates subsequently altered their social associations, and examine the extent to which the social consequences of the removals depended on the individual's prior social connection to the removed birds. Finally, we show that removed birds largely regained their previous social associations upon returning to the wild. We discuss the significance of these findings for understanding how natural populations respond to the loss of individuals, the mechanisms driving these responses, and their potential applied implications.

## 2. Material and methods

### (a) Study system

This study was conducted on a long-term study population of great tits in Wytham Woods, Oxford, UK (51°46′ N, 1°20′ W), where breeding adults and their chicks have been marked with unique BTO (British Trust for Ornithology) metal leg rings since the 1960s [39]. Since 2007, all captured great tits have also been fitted with plastic leg bands containing RFID microchips. From September to February (non-breeding season), great tits aggregate in winter foraging flocks [40] and extensive mist netting is carried out to also mark immigrant birds with RFID tags. These tags allowed the detection of the time, date and location of each individual's presence at sunflower seed feeding stations equipped with RFID antennae attached to two opposing access holes (Dorset ID, Aalten, The Netherlands). These were placed in a stratified grid at 65 fixed locations, approximately 250 m apart throughout Wytham Woods, and opened automatically every weekend over the winter, scanning for RFID tags from pre-dawn until after dusk.

#### (i) Inferring social networks

Detections of RFID tags at the feeding stations provide a fine-scale temporal datastream made up of bursts of activity as flocks arrive and feed [41,42]. These ‘flocks’ (or ‘flocking events’) can be identified using a machine learning algorithm which employs a Gaussian mixture model (GMM) to assign each individual detection to the event it is most likely to have occurred in [41]. This provides an objective way of identifying flocks, and is preferable to applying techniques requiring the specification of arbitrary parameters, such as set time windows, to define co-occurrences [42].

By calculating the flock co-memberships among all individuals, we created a ‘group-by-individual’ matrix [9], specifying co-occurrences between all birds. We used R v. 3.2.2 [43] for all analyses, and applied the simple ratio index (SRI) [44] to calculate weighted social associations among individuals to create social networks. In this way, the network ‘nodes’ represent the individual birds, while the ‘edges’ linking them represent the dyadic social associations. Social networks within this system are known to be non-random after accounting for spatial structure, to carry over across contexts and to be important to various social processes [16–18,25,45–49]. We created social networks for each weekend separately. Further, previous work has indicated that pooling data over longer time periods can provide a more accurate depiction of individuals' social phenotypes than single sample periods [25]. Therefore, for each weekend, we also calculated cumulative networks by considering all flocking events recorded from the beginning of the season up to the end of the focal weekend, creating a social network from all possible data available at that time.

### (b) Experimental procedure

**Figure 1.**
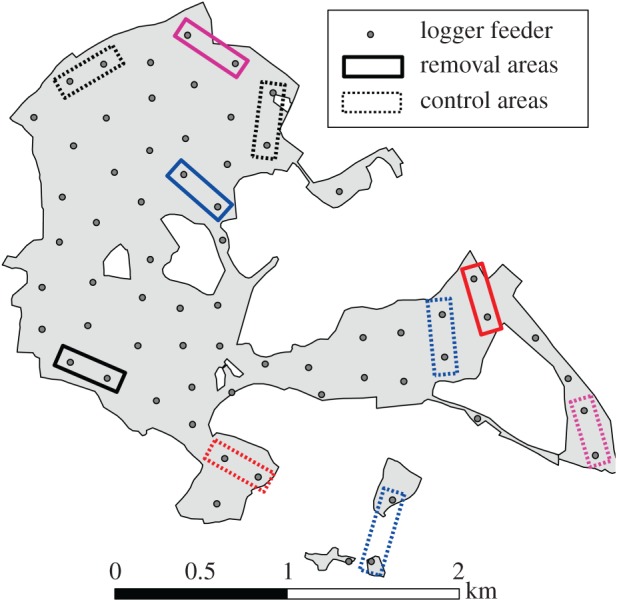
Wytham Woods, Oxford, UK, with RFID feeding stations shown as grey circles. Rectangles show areas where the removal treatment/catching control was carried out, where the same colours represent the same replicate. These took place in order of red, black, blue, purple. Areas where birds were captured and immediately released (control areas) are boxed in dotted lines and areas where birds were subjected to removal (removal areas) are in solid lines.

The experiment began in September 2013, four weeks into standard winter data collection. Four replicates were carried out, with one week between each replicate. All experiments followed the same standard protocol. In each replicate (i.e. each week), two neighbouring feeding stations (hereafter referred to as an ‘area’) were chosen for the removals (figure 1). To ensure that removals were feasible, we used previous logging data to choose an area with relatively high numbers of birds. Removals were carried out using standard mid-week winter mist-netting, which was carried out at the chosen feeding stations. In each replicate, we aimed to capture and remove six RFID-tagged great tits. On one occasion, 12 birds were caught so six were randomly selected, while in another replicate, only five were caught and removed. Removed birds were then held in captivity over the weekend logging period, and released the following Monday. The experimental period spanned four weeks, took place over four areas (two neighbouring feeding sites in each) and consisted of temporarily removing 23 RFID-tagged great tits in total.

During the experimental period, we also carried out controls (figure 1). This consisted of carrying out standard mid-week catching sessions of similar intensity (approx. 3.5 h) at areas (each also consisting of paired feeding stations) that had a similar number of birds to the concurrent removal sites but minimal exchange of individuals between them. During the control catching sessions, all birds were released within 30 min after capture. Over the experiment, we alternated whether catching took place first at the removal or the standard capture areas. Entirely standardized effort and matching under the field conditions was impossible due to variation in weather conditions and environmental surroundings changing the number and ease of catching individuals. However, from the onset of data collection to the end of the experiment (but excluding the experimental weekends at each site), a similar number of birds were recorded each weekend in the removal (mean ± s.e.: 24.00 ± 2.2) and control (21.0 ± 1.3) areas, with no significant difference based on treatment assignment (linear model controlling for time and replicate: *t* = 1.01, *p* = 0.32). Further, the turnover of birds (i.e. number of birds remaining in an area divided by the total number of birds recorded over both weekends) was also not significantly related to treatment assignment (removal = 0.54 ± 0.03, control = 0.52 ± 0.04, linear model: *t* = 0.02, *p* = 0.98). Carrying out the standard capture sessions (without removal) in matched areas ensured we were subsequently able to compare the effects of experimentally imposed loss of individuals with any underlying effects produced simply by the mist-netting procedure.

### (c) Assessing the experimental effects

For each trial, the focal individuals were defined as those recorded in the weekend directly before and after the trial. We refer to birds recorded in the same flock before the trial as ‘flockmates’. Across the woodland, focal individuals whose flockmates were not captured or removed were categorized as ‘non-affected’. The flockmates of birds who were removed were categorized as having their ‘flockmate removed’, while the flockmates of birds captured at the control sites were categorized as having their ‘flockmate captured’. No birds fell into multiple categories during a trial. Birds which were actually captured/removed during the trial were not considered as focal individuals. Furthermore, when assessing changes in social associations in response to the experiment (see below), captured/removed birds' associations to others were excluded to allow us to compare the control and removal treatment in a relevant way.

The effects of the removals may be related to individuals' number and strength of social associations towards the removed birds. For example, birds experiencing the removal of a single, weakly associated flock member should be affected less than those who lost six flock members with which they held strong social associations. Therefore, we calculated the proportion of each focal individual's total social associations (i.e. total ‘strength’—the sum of their edge weights) in the week before the trial that was directed towards subsequently removed individuals (or directed towards captured and released individuals in the controls). We refer to this measure as the extent of the ‘social impact’ experienced. We then examined how this social impact predicted changes in individuals' associations.

#### (i) Assessing changes in behaviour and social associations

As changes to individuals' general foraging behaviour could potentially indirectly influence their social associations, we first considered three basic measures of foraging behaviour. These were individuals' average (i) raw activity (i.e. second-by-second detections) at the feeders, (ii) number of flocking events (i.e. output of the GMM) and (iii) number of feeding sites visited. We then assessed four social metrics that are known to be repeatable within individual great tits over weeks and years, even when accounting for space use [25]. We calculated each individual's (i) average flock size, (ii) number of unique flockmates (i.e. unweighted degree), which represents their general gregariousness, (iii) the sum of all their social associations (i.e. strength), which measures their general network centrality, and (iv) ‘betweenness’ (i.e. the number of shortest paths between other individuals in the network that pass through the individual), which represents how an individual might act as a bridge for transmission of information and disease [10]. Betweenness was log-transformed to reduce skew [25]. Finally, we assessed (v) the average score of their dyadic social associations to each of their flockmates (i.e. average edge weight), which indicates the tightness of their social bonds [50].

We assessed how each focal individual's social metrics changed following the trial (i.e. immediately ‘post-trial’) in relation to their pre-trial metrics. Pre-trial networks were calculated from all of the weekend network data recorded before the trial took place (but see the electronic supplementary material for alternative analysis). However, as unweighted degree is intuitively expected to be larger in cumulative networks than any stand-alone sampling period, we calculated pre-trial unweighted degree as birds' average degree score over the previous weekends. As network parameters are likely to change even over short periods due to the dynamic and variable nature of the fission–fusion system [45], we expressed each individual's change as relative to the average change over all individuals over this time-frame.

#### (ii) Assessing the relationship between social loss and change in metrics

We primarily aimed to assess whether the social impact of the removals caused changes in individuals' social associations over and above that expected. Thus, for all individuals that had a flockmate removed/captured in a trial, we assessed how subsequent changes in behaviour were predicted by (i) treatment category (i.e. whether their flockmates were subjected to just standard capture [control] or removal), (ii) the proportion of their social associations to these removed/captured flockmates (i.e. ‘social impact’), and, importantly, (iii) the interaction of these two predictors. The interaction term allows the separation of whether behaviour changes are due to the extent of social loss they experience or simply due to exposure to the procedure whereby flockmates are captured. Therefore, we ran linear mixed models (LMMs) for each social metric (response variable). The predictor variables were set as the treatment category, the social impact, and the interaction between these. We included trial week as a fixed effect to account for temporal changes, and set individual identity as a random effect.

Owing to the implicit non-independence of network data, we also used simple network permutations to derive *p*-values by comparing the *t*-value of each model's coefficients to those generated from 10 000 node randomizations [9,51]. For each trial, the permutations randomly reassigned each individual to another individual's pre- and post-trial network position. This maintained the same network structure and distribution of changes in metrics, but randomized the treatment category that each within-individual change was assigned to within each trial.

#### (iii) Reintroductions

While our main goal was to determine how individuals respond to the loss of social associates, we were also able to examine whether individuals changed their behaviour upon the reintroductions of removed birds (as removed birds were reintroduced after one weekend). Therefore, we used the same structure models described above, but instead of setting the response variables as individuals' change in social metrics immediately following the trial, we considered their change in social metrics following the reintroductions. In this way, we tested whether prior social associations towards removed birds related to changes in individuals' social metrics upon the reintroduction of their flockmates.

Further, we also examined whether reintroduced birds regained their social associations to their previous flockmates. First, we tested if removed birds' previous social associations towards other birds occuring at the same feeders as them (prior to their removal) predicted whether they would be flockmates following reintroduction using a GLMM with binomial error structure and logit-link function. We included replicate number, removed individuals' time until resiting and distance from initial capture site upon resiting as fixed effects, and set individuals' identities as random effects. We then used the same structure GLMM to assess whether removed individuals' pre-removal dyadic association strength to their flockmates (i.e. only considering those observed in a pre-trial flocking event with them) predicted their dyadic association strength following reintroduction. In this case, dyadic association strength was modelled as the number of flocks the dyad co-occurred (successes) in relation to the total number of flocks the dyad did not co-occur (fails) as a binomial equivalent of the SRI.

## 3. Results

Over the woodland during the main eight-week study period, 395 113 records of 542 unique great tits making up 18 388 flocks were recorded. The flock size experienced by the average individual (i.e. ‘typical’ group size [52]) was 4.86 ± 0.02 (mean ± s.e.). The number of focal individuals in each trial (i.e. those observed in sampling periods directly before and after the trial but not captured themselves) ranged from 15 to 49 for those having a flockmate removed, 7 to 46 for those having a flockmate captured and immediately released (i.e. control treatment), and 152 to 192 for those whose flockmates were unaffected. The average network each weekend contained 307 individuals (range: 287–352) with an edge (social connection) density of 3.86% (range: 3.5–4.2%). On a weekend-to-weekend basis, the social network remained highly consistent as individuals largely maintained their dyadic associations to others (weekend-to-weekend Mantel test range: *r* = 0.65–0.78, *p* < 0.0001).

### (a) Effect of removals on foraging behaviour of flockmates

We found no differences between birds whose flockmates were captured (control), birds that experienced flockmate removal and birds whose flockmates were unaffected (LMMs, all category factor *t* < 1.8, *p* > 0.06; electronic supplementary material, figure S1) in their change in the number of flocks they occurred in and number of sites they visited. Birds that experienced flockmate removal showed increased activity at the feeding stations (LMM, removal category factor *t* = 2.5, *p* < 0.05). However, the interaction between treatment category (i.e. whether an individual experienced flockmate loss, or flockmate capture) and social impact (proportional association strength to removed/captured flockmates) did not predict changes in any measures of foraging behaviour (LMM, *t* < 0.76, *p* > 0.45; table 1*a–c*). Therefore, while birds which fell into the ‘flockmate removed’ category showed a small increase in activity at feeding stations, the extent of loss of social associates had no influence on any of these behaviours above that expected by the capture procedure alone.

**Table 1.** Results of full models corresponding to figure 2*f–j*. LMMs all included individual identity as random effects and assessed the effect on the response variable (change in the social metric) by the fixed effects of (i) ‘prop. assoc’ (i.e. the proportion of an individual's association held to removed/captured individuals), (ii) ‘treatment’ (i.e. whether the individual's flockmates were just captured or actually removed), (iii) the week in which the replica took place, and (iv) the interaction between ‘prop. assoc’ and ‘treatment’. The coefficient, standard error, *t*-value and the standard *p*-value are provided, along with the *p*-value calculated from the randomizations (*p*_rand_).

		coeff.	s.e.	*t*	*p*	*p*_rand_
(*a*) change in no. records	intercept	−11.496	22.234	−0.517	0.606	0.584
	prop. assoc	55.479	63.729	0.871	0.39	0.411
	treatment	6.363	23.915	0.266	0.792	0.806
	week	3.162	6.673	0.474	0.639	0.576
	interaction	65.683	111.49	0.589	0.559	0.535
(*b*) change in no. flocks	intercept	−2.304	2.686	−0.858	0.392	0.352
	prop. assoc	8.084	7.797	1.037	0.307	0.283
	treatment	0.808	2.819	0.287	0.776	0.787
	week	0.497	0.801	0.621	0.539	0.462
	interaction	5.544	12.922	0.429	0.67	0.652
(*c*) change in no. sites visited	intercept	−0.03	0.096	−0.31	0.757	0.751
	prop. assoc	−0.224	0.279	−0.803	0.427	0.493
	treatment	−0.148	0.1	−1.485	0.146	0.141
	week	0.042	0.029	1.48	0.148	0.121
	interaction	0.339	0.453	0.75	0.458	0.467
(*d*) change in flock size	intercept	−0.108	0.421	−0.257	0.798	0.794
	prop. assoc	−1.497	1.224	−1.223	0.229	0.226
	treatment	−0.591	0.445	−1.326	0.193	0.187
	week	0.22	0.126	1.748	0.089	0.036
	interaction	2.771	2.053	1.35	0.185	0.171
(*e*) change in degree	intercept	−1.479	1.603	−0.923	0.357	0.326
	prop. assoc	−0.14	4.675	−0.03	0.976	0.953
	treatment	−2.785	1.678	−1.66	0.106	0.097
	week	1.006	0.48	2.096	0.043	0.014
	interaction	15.233	7.652	1.991	0.054	0.051
(*f*) change in strength	intercept	−0.169	0.222	−0.762	0.447	0.411
	prop. assoc	−0.339	0.643	−0.527	0.602	0.56
	treatment	−0.244	0.237	−1.029	0.31	0.318
	week	0.094	0.067	1.411	0.167	0.099
	interaction	2.999	1.098	2.732	0.01	0.009
(*g*) change in betweenness	intercept	−0.596	0.572	−1.043	0.298	0.284
	prop. assoc	1.539	1.659	0.927	0.36	0.402
	treatment	1.156	0.607	1.903	0.065	0.059
	week	0.141	0.171	0.822	0.417	0.34
	interaction	−5.412	2.808	−1.928	0.062	0.062
(*h*) change in average edge weight	intercept	0.014	0.013	1.06	0.291	0.267
	prop. assoc	−0.061	0.039	−1.589	0.121	0.08
	treatment	0.005	0.014	0.355	0.725	0.716
	week	−0.008	0.004	−2.063	0.046	0.036
	interaction	0.123	0.063	1.968	0.057	0.043

### (b) Effect of removals on social behaviour of flockmates

**Figure 2.**
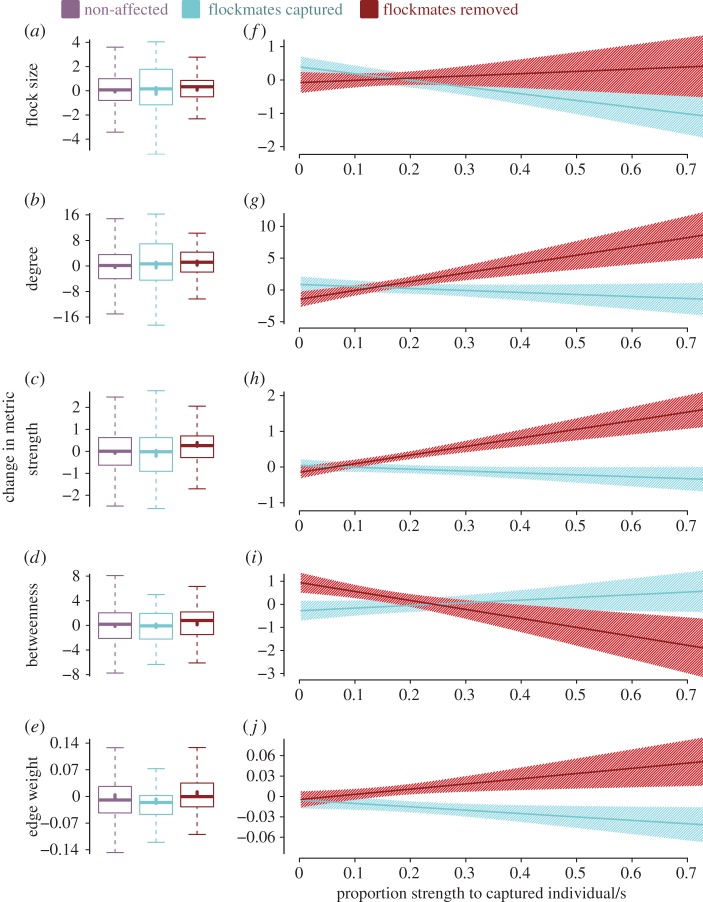
Change in great tit social network metrics under the different treatment conditions of (i) not affected (purple), (ii) flockmates captured and immediately released (blue), and (iii) flockmates removed (red). (*a–e*) The raw data expressed with boxplots showing the change in the social metrics for individuals in each category. Thick vertical lines show mean ± s.e., mid-lines show median, box shows interquartile range (IQR), whiskers shows range (with values outside 1.5 times IQR excluded). (*f–j*) Models assessing how individuals' subsequent changes in social network metrics (*y*-axes) are related to their proportion of previous social association strength that was either (i) directed towards removed flockmates (the red lines show individuals who experienced the experimental treatment of flockmate removal) or (ii) directed towards flockmates which were captured and immediately released (the blue lines show individuals who experienced the control treatment of flockmate capture). Lines and surrounding shaded area show LMM fit and standard error over all replicates (table 1*d–h* for full model details).

Among the treatment categories (i.e. (i) no treatment, (ii) flockmate capture and immediate release and (iii) flockmate removal), the only difference found in social metrics was that those who experienced flockmate removal had, on average, a slightly higher increase in network strength compared with birds whose flockmates were either just captured or not affected at all (permutation test, *p* < 0.01; figure 2*a–e*). Thus, treatment group alone had little influence on the change in individuals' social network metrics (figure 2*a–e*).

Importantly, by assessing the interaction between treatment category and the social impact experienced, we determined how the extent of the social loss an individual experienced affected individuals' social metrics. We found that relative changes in average flock size were not significantly predicted by this interaction (figure 2*f* and table 1*d*). Therefore, the extent of social loss did not affect this relatively simple measure of sociality more than expected from any disturbance caused by the capture procedure alone. However, when considering individuals' social network metrics, we found that the interaction between treatment class and social impact significantly predicted changes in average unweighted degree and strength (figure 2*g,h* and table 1*e,f*). Flockmates of birds that were just captured showed no change in their social associations with increased social impact, but those birds that lost flockmates showed increases in degree and strength with increasing levels of previous social association to the removed birds (figure 2*g,h*).

Therefore, those experiencing the most social impact in terms of removing their previous flockmates showed the greatest increases in their degree and strength to remaining individuals. For instance, around one-third of birds that experienced flockmate removal only lost a small proportion (less than 10%) of their network connection strength (i.e. summed edge weights), and these generally showed very little change in the number of flockmates (figure 2*g*). Yet around one-fifth of birds experiencing flockmate removal lost more than 50% of all their previous network connection strength and generally increased their number of connections to the remaining individuals by five (figure 2*g*). Those suffering this large social impact also increased their overall network strength by approximately 1 (figure 2*h*). This increase, for example, roughly equates to spending an additional 20% of their time with five flockmates who also spend an additional 20% of their time with them. Indeed, the general increase in network strength was not entirely due to increasing their number of new social associates, as individuals experiencing the most social loss from removals also showed an additional increase in the tightness of their social connections (average edge weight; figure 2*j*). This is demonstrated by the interaction between treatment category and social impact having a significant effect on relative change in average edge weight (table 1*h*—but note marginally non-significant when not using permutations).

We also assessed ‘betweenness’, which is more complex than the other social network metrics as it considers associations among all individuals, even between those not directly associated with the focal individual and therefore might depend less on the focal individual themselves [12]. We found that the social loss an individual experienced did not cause changes in betweenness, as there was no significant interaction between treatment category and social impact (figure 2*i* and table 1*g*).

### (c) Construct validity

As social network metrics can be inferred/derived in various ways, we assessed whether our findings that individuals increased their social connectivity in response to experiencing social loss were validated under alternative analysis (see the electronic supplementary material for details). We found the same patterns of effects of social loss on social association metrics when using a more basic measure of social impact (proportion of flockmates removed/captured) (electronic supplementary material, figure S2*a–e*) and when using averaged scores of social association metrics (electronic supplementary material, figure S2*f–j*). The estimated statistical significance of the model parameters differed slightly among the three approaches (table 1; electronic supplementary material, table S1), but the primary approach is expected to be the most reliable due to the higher resolution of both the estimate of social impact (i.e. using strength rather than degree) and of the response variables (i.e. using cumulative data rather than averaged networks). We also found that before the experiments took place, individuals' weekly change in their social network metrics (i.e. the response variables of interest) was unrelated to whether or not they subsequently experienced removal or capture of their flockmates and the subsequent ‘social impact’ they would experience (electronic supplementary material, figure S3*a–e*). Therefore, individuals' increases in degree, strength and average edge weight in response to experimentally imposed flockmate loss do not appear to be driven by any pre-existing differences between individuals or sites, but instead caused by the removals.

### (d) Reintroductions

As 20 out of 23 removed individuals were resited after reintroduction, we were additionally able to examine the effect of the reintroductions. Upon release, 85% of resited reintroduced birds were first detected on the same, or neighbouring, feeding station that they were removed from. In 80% of occasions, this was on the first weekend following their release. We first considered how birds that had experienced flockmate removal responded upon the reintroduction of their flockmates. We found that pre-trial social associations towards removed birds did not relate to changes in social association metrics upon the reintroduction of the removed birds (electronic supplementary material, figure S3*f–j*).

**Figure 3.**
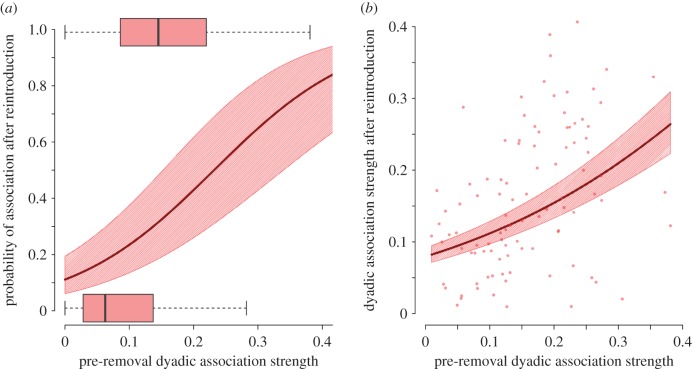
The recovery of previous social associations upon the reintroduction of removed birds. (*a*) Removed birds' previous dyadic social associations with other birds occurring at the same feeders as them (*x*-axis) predicted whether they were flockmates following reintroduction (*y*-axis) and (*b*) removed birds' previous dyadic social associations with their previous flockmates (*x*-axis) predicted their dyadic association strength with them following reintroduction (*y*-axis). In both panels, solid lines and surrounding hashed area show fit and standard error, respectively, of GLMMs including individuals' identities as a random effect (see electronic supplementary material, table S2 for full model results). Boxes in (*a*) show the raw data interquartile range (IQR), with mid-lines denoting median and whiskers indicating the range (excluding values 1.5 times outside of IQR). Points in (*b*) show raw dyadic social association strengths between removed birds and their flockmates. (Online version in colour.)

We then considered the reintroduced birds themselves, and found these generally regained the same social associates upon reintroduction. Removed birds' social associations towards other birds occurring at the same feeders as them prior to their removal strongly predicted whether they would be flockmates following reintroduction (GLMM: estimate = 8.4 ± 2.1, *z* = 3.926, *p* < 0.001—figure 3*a*; electronic supplementary material, table S2*a*). Further, the pre-removal social association strength of reintroduced birds to their previous flockmates strongly related to the post-reintroduction association strength to the same flockmates (GLMM: estimate = 3.8 ± 0.4, *z* = 8.659, *p* < 0.001—figure 3*b*; electronic supplementary material, table S2*b*).

## 4. Discussion

By temporarily removing individuals from a social network of wild great tits, we demonstrated experimentally that the loss of conspecifics has significant social ramifications for the remaining individuals. Experiencing the loss of social associates caused birds to form new associations with others, increase their general connectedness within the social network and form tighter social ties to their remaining flockmates (figure 2 and table 1). The changes were particularly striking as they appear to be driven by fine-scale modifications of social association patterns in response to loss of their flockmates, rather than by changes to individuals' general foraging behaviour (table 1*a–c*; electronic supplementary material, figure S1).

These findings represent an important contribution to understanding the social consequences of losing individuals from wild animal societies, as previous knowledge has been largely based on computer simulations [29–31,35,53]. Although simulations could potentially be applied to a variety of systems, any such findings are currently difficult to interpret given the lack of understanding regarding biological systems' responses to perturbations [33–35]. In particular, studies on captive animals have demonstrated how individuals might react to social loss in ways that would be difficult to account for using simulations alone [36,37]. Through demonstrating that individuals experiencing loss of their associates showed increased social associations to the remaining individuals, we illustrate the importance of considering behavioural responses to social loss in wild populations.

In natural settings, gain and loss of individuals (due to birth/deaths, or migration/immigration) may be expected to continually alter individuals' interaction partners. Even with such turnover, remaining individuals may maintain consistent social network positions. For example, great tits express repeatable social phenotypes over years despite approximately 50% annual turnover [25]. Our findings, indicating an upregulation of other social associations in response to loss, potentially illustrate the means by which individuals demonstrate repeatable social phenotypes, despite a continually changing pool of interaction partners. As great tits naturally experience reasonably high levels of social turnover, the evolution of strategies to buffer their own patterns of social associations against perturbation may be expected in comparison with species experiencing less social mixing. Indeed, if social network connections influence fitness [19–21], repeatable differences between individuals create the potential for selection to shape the social network structure [54].

The biological mechanisms underpinning individuals' responses to social perturbations have been relatively unexplored. In this study, the fact that birds appear to increase their general sociability upon losing their associates may represent a rapid behavioural response to compensate for the loss of connectedness. Social associations are known to be valuable to individuals [55]. For instance, within our study population, the most central individuals benefit from increased access to information regarding new food sources [16]. Thus, while the loss of flockmates may reduce an individual's access to information, rapidly acting to increase their centrality may mitigate this. In the same sense, previous experiments that separated flockmates from foraging together [47] indicated that birds can increase their usage of social information from heterospecifics in response to social segregation [18].

Maintaining high numbers of flock members can also help protect against predation [55], and, within this study system, simulated predation risk has been shown to increase flock turnover, potentially causing individuals to form more social associations [56]. Therefore, it could be hypothesized that increases in social centrality with increasing amounts of flockmate removal (but not simply with increasing exposure to the capture procedure) are due to birds recognizing the actual loss of their associates, and interpreting this as a cue of high predation conditions (thus causing them to favour central network positions). In this case, however, changes to other foraging behaviours linked to anti-predator responses, such as activity and movements, would also be expected. Yet none of these foraging behaviours were strongly related to the extent of flockmate loss. Indeed, along with short-term benefits (such as predation avoidance), the future benefits of sustaining associations may be as important, or more important. For instance, pair members remain strongly associated through the winter and base their behaviour around one another [49]. Birds also appear to shape their breeding positions and territories around their close winter associates, potentially to reduce competition and increase cooperation during breeding [46].

Rather than a compensatory response to losing social associations, an alternative hypothesis for the increase in centrality measures may be competitive replacement. Early, seminal studies of great tits found that breeding territories and locations were limited, and removing individuals from their territory resulted in rapid replacement by close neighbours from non-optimal territories [1]. Similarly, for various tit species, removed winter groups appear to be quickly replaced by new groups [57]. Although attaining and maintaining certain network positions might require considerable input from individuals [54], it is unclear whether winter social network positions are ‘limited’. Such limitations could occur if, for example, social centrality was a desired attribute, and individuals generally aimed to associate with the most ‘attractive’ individuals. In this way, ‘attractive’ individuals would be able to hold the most social connections (as others are attracted to associating with them), and therefore the removal of individuals may ‘free up’ connections to be adopted by those socially closest to them, and result in the pattern of increased centrality with increased social loss (figure 2).

Ultimately, developing a better understanding of individuals' responses to loss will improve the ability to predict the consequences. For example, a recent observational study indicated that wild African elephant (*Loxodonta africana*) populations maintain their social network structure, despite ivory poaching eliminating the highly connected nodes (i.e. older female elephants) [58]. While no active response to such loss was exhibited, the robustness instead stemmed from daughters replicating their mothers' social positions. This generated social redundancy, and allowed structural maintenance upon the removal of the mother [58], resulting in resilient connected groups. Therefore, while one might expect that poaching of elephant matriarchs would reduce the availability of important information to the rest of the group [59], the expected loss in social network connectivity was mitigated through this underlying resilience.

The resulting social structure following individual loss also has important implications for predicting infectious disease spread [60]. For instance, culling Tasmanian devils (*Sarcophilus harrisii*) known to be carriers of an infective facial tumour disease did not reduce its spread, as the highly connected social system lead to rapid transmission regardless of relatively small-scale losses. Similarly, attempts to control rabies within vampire bats (*Desmodus rotundus*) through removing adults were found to be ineffective as younger individuals were the primary transmitters [61]. Thus, although procedures that actively identify and remove highly connected individuals may aid in reducing disease spread, our findings caution that this too may be less effective than predicted if the remaining individuals increase their social connections in response to the removals.

Various ecological studies also rely on procedures that (temporarily) remove individuals from wild populations (such as for marking, behavioural assaying or short-term captive experiments). The consequences of this disturbance for study systems are generally unknown. We demonstrate that removals can have social impacts that would otherwise go unnoticed, particularly as individual-level activity patterns remain mostly unchanged (electronic supplementary material, figure S1). The effect of reintroducing individuals into populations is also rarely considered, but can also influence social behaviour [62]. Here, we show that individuals experienced little interference upon the reintroduction of their flock mates (electronic supplementary material, figure S3*f–j*). Interestingly, reintroduced individuals generally regained their prior social network connections (figure 3; electronic supplementary material, table S2). This indicates that social associations between great tits may be resilient to short-term separations and perturbations. Similarly, the social structure of captive guppy shoals exhibited resilience to the reintroduction of individuals following their removal during a cooperative task [63], which also suggests maintenance of associations despite such disturbance. On the other hand, consequences of introductions for captive catsharks (*Scyliorhinus canicula*) appear to depend both on the type of individual introduced and the characteristics of the social group [64]. Thus, further examination of the social consequences of the addition of individuals within wild populations appears to be a useful avenue of future research [62].

## Supplementary Material

Supplementary Information

## 5. Conclusion

We provide experimental evidence that the removal of individuals from wild populations has social implications that expand beyond the direct effects of social associate loss. Individual great tits responded to increasing amounts of externally imposed flockmate loss by increasing their social connectivity to others. This demonstrates that reductions in connectedness within the social network itself due to individual loss may be ameliorated by increases in associations between remaining animals. Therefore, along with increasing our knowledge of the stability of social organization, these findings may have implications for predicting how individual loss can alter social structure and social processes important to conservation efforts, such as the spread of disease or information between individuals. Future work examining the generality and untangling the mechanisms of this compensatory response to social loss, along with experiments varying both the quantity and type of experimentally removed individuals across a range of social systems, would be particularly valuable.

## Data accessibility

Supporting data are freely available at: http://dx.doi.org/10.5061/dryad.q8987 [66].
