# Supplementary material for: Wild birds respond to flockmate loss by increasing their social network associations to others
Source: Proc Biol Sci. 2017 May 17;284(1854):20170299. doi: 10.1098/rspb.2017.0299 (PMC5443949; doi:10.1098/rspb.2017.0299)
Supplement: Supplementary Information [file rspb20170299supp1.docx]

**SUPPLEMENTARY INFORMATION: Wild birds respond to flockmate loss by increasing their social network associations to others**

Article DOI: 10.1098/rspb.2017.0299

Josh A. Firth^1^*, Bernhard Voelkl^1,2^, Ross A. Crates^1,3^, Lucy M. Aplin^1^, Dora Biro^4^, Darren P. Croft^5^, Ben C. Sheldon^1^

*^1^Edward Grey Institute, Department of Zoology, University of Oxford, Oxford, OX1 3PS, UK*

*^2^Animal Welfare Division, Vetsuisse Faculty, University Bern, Laenggassstrasse 120, 3012 Bern, CH*

*^3^Fenner school, Australian National University, Canberra, Australia 2601.*

*^4^Department of Zoology, University of Oxford, Oxford, OX1 3PS, UK*

*^5^Centre for Research in Animal Behaviour, College of Life and Environmental Sciences, University of Exeter, Exeter EX4 4QG, UK*

*Correspondence: Joshua.firth@zoo.ox.ac.uk

***Supplementary analysis***

The primary analysis consisted of determining how the amount of social loss an individual experienced related to subsequent changes in their social network metrics. However, due to the nature of inferring social networks, the predictor variables of interest (‘social impact’) and the response variables (change in social network metrics) can potentially be scored in various ways. Therefore, we aimed to ensure that any experimental effects identified from the primary analysis were robust to the various ways that social network parameters can be quantified.

Firstly, instead of considering ‘social impact’ as the proportion of all their weighted social associations directed towards removed individuals, we instead set this predictor variable simply as the proportion of flockmates removed/captured (Figure S2a-e). This represents a very basic measure of social impact and is equivalent to the proportion of their total binary connections in the previous week directed towards captured individuals (i.e. the unweighted version of the primary analysis).

Secondly, the primary analysis used all prior network data to calculate individuals’ pre-trial social association metrics, as previous work has suggested that cumulative networks provide the best representation of individuals’ social network metrics (Aplin et al. 2015). However, we also carried out supplementary analysis that considered individuals’ social metrics as an average calculated from social networks built for each pre-trial weekend separately (i.e. applying the same methodology used to calculate changes in the ‘degree’ metric - see *Methods*). Therefore, we repeated the models evaluating changes in flock size, strength, betweenness, and edge weight, but set pre-trial score as their average score over previous weekly networks (Figure S2f-j).

Third, the primary analysis assumes that birds from control areas do not intrinsically differ from those in removal areas. Indeed, control areas did not differ from removal areas in the number of birds or turnover of individuals recorded at the feeding stations (see *Methods*). However, we additionally assessed whether any prior differences in the primary variables of interest existed between those birds that were associated to subsequently removed birds and those associated to the birds that were captured and released. Therefore, we repeated the primary models but considered individuals’ behavioural changes in weeks before the experiments even took place, with the prediction that no differences between flockmates of subsequently removed or control-capture birds would exist (Figure S3a-e).

**Figure S1.** Change in metrics of basic foraging behaviour given treatment condition (not affected, flockmate/s captured, or flockmate/s removed). Parts a-c show boxplots of change in metrics for those who fall into each category. Thick vertical lines show mean±SE, mid horizontal lines show median, box shows interquartile range (IQR), whiskers shows range (with values outside 1.5 times IQR excluded). Parts d-f shows models assessing how individuals’ subsequent changes in these non-social network metrics (y-axes) are related to their proportion of previous social association strength that were either (i) directed towards removed flockmates (the red lines show individuals who experienced the experimental treatment of flockmate removal) or (ii) directed towards flockmates which were captured and immediately released (the blue lines show individuals who experienced the control treatment of flockmate capture). Lines and surrounding polygons show LMM fit and standard error over all replicates (see Table 1a-c for full model details). Over all measures of changes in foraging behaviour, there was no significant interaction between treatment class (flockmates removed or captured) and social impact. There was also no significant effect of previous social association towards removed/captured flockmates regardless of treatment type, but there was a weak, non-significant, trend for those with stronger associations to removed/captured flockmates to show a relative increase in their activity at feeding stations (Table 1a-b).

**Figure S2.** Change in social behaviour given treatment condition (red lines=flockmate/s removed, blue lines=flockmate/s captured and released). Follows Figure 2 in main text, but presents results when proportion of flockmates captured is used for the primary predictor variable (a-e) and when the response variable is calculated as the change in comparison to the pre-trial metrics weekly average (f-j). Sections show individuals’ change in that social metrics over the proportion of their social associations directed towards captured/removed individuals (see above). Lines and surrounding polygons show LMM fit and standard error over all replicates (see Table S1 full model details).

**Figure S3**. Change in social behaviour given treatment condition (red lines=flockmate/s removed, blue lines=flockmate/s captured and released). Follows Figure 2 in main text but considers different time periods. (a-e) Individuals’ change in social behavior the week before the experiment is not predicted by an interaction between social impact (x axis) and treatment condition (flockmates captured or removed). (f-j) Individuals’ change in social behavior following the reintroduction of the removed birds is not predicted by an interaction between social impact (x axis) and treatment condition (flockmates captured or removed). Sections show individuals’ change in that social metrics over the proportion of their social associations directed towards captured/removed individuals (see above). Lines and surrounding polygons show LMM fit and standard error over all replicates.

**Table S1.** Results of full model corresponding to Figure S2. The table follows Table 1 in main text, but presents results when (a) proportion of flockmates captured/removed is used for the primary predictor variable (“proportion of degree to captured”) and when (b) change from the pre-trial weekly average of each metric is used as the response variable. LMMs all included individual identity as random effects and assessed the effect on the response variable of the change in the social metric by the fixed effects of (i) ‘Prop. affect’, which is the proportion of individuals’ associations held to captured/removed individuals (see above) (ii) ‘Treatment” i.e. whether the individuals’ flockmates were just captured or removed, (iii) the week which the replica took place and (iv) the interaction between ‘Prop. assoc’ and ‘Treatment’. The coefficient, standard error, t value and the standard p value are provided, along with the p value calculated from the randomisations (P_rand_).

|  |  | a) Proportion of degree to captured/removed | | | | | b) Change from pre-trial weekly average | | | | |
| --- | --- | --- | --- | --- | --- | --- | --- | --- | --- | --- | --- |
|  |  | Coeff. | SE | t value | P | P_rand_ | Coeff. | SE | t value | P | P_rand_ |
| Change in Flock size | Intercept | -0.185 | 0.405 | -0.457 | 0.648 | 0.607 | -0.108 | 0.421 | -0.257 | 0.798 | 0.784 |
|  | Prop. affect | -1.271 | 1.189 | -1.069 | 0.292 | 0.289 | -1.497 | 1.224 | -1.223 | 0.229 | 0.231 |
|  | Treatment | -0.632 | 0.523 | -1.209 | 0.235 | 0.209 | -0.591 | 0.445 | -1.326 | 0.193 | 0.172 |
|  | Week | 0.227 | 0.127 | 1.781 | 0.083 | 0.028 | 0.22 | 0.126 | 1.748 | 0.089 | 0.031 |
|  | Interaction | 3.22 | 2.784 | 1.157 | 0.255 | 0.214 | 2.771 | 2.053 | 1.35 | 0.185 | 0.16 |
| Change in Degree | Intercept | -1.612 | 1.544 | -1.044 | 0.298 | 0.241 | -1.479 | 1.603 | -0.923 | 0.357 | 0.307 |
|  | Prop. affect | -0.554 | 4.545 | -0.122 | 0.904 | 0.891 | -0.14 | 4.675 | -0.03 | 0.976 | 0.971 |
|  | Treatment | -4.225 | 1.949 | -2.168 | 0.037 | 0.028 | -2.785 | 1.678 | -1.66 | 0.106 | 0.107 |
|  | Week | 1.122 | 0.484 | 2.318 | 0.026 | 0.004 | 1.006 | 0.48 | 2.096 | 0.043 | 0.01 |
|  | Interaction | 24.702 | 10.284 | 2.402 | 0.022 | 0.014 | 15.233 | 7.652 | 1.991 | 0.054 | 0.051 |
| Change in Strength | Intercept | -0.205 | 0.213 | -0.963 | 0.337 | 0.277 | -0.29 | 0.217 | -1.337 | 0.183 | 0.15 |
|  | Prop. affect | -0.203 | 0.618 | -0.329 | 0.744 | 0.735 | -0.479 | 0.633 | -0.757 | 0.454 | 0.447 |
|  | Treatment | -0.414 | 0.28 | -1.479 | 0.148 | 0.136 | -0.258 | 0.226 | -1.14 | 0.262 | 0.266 |
|  | Week | 0.1 | 0.067 | 1.483 | 0.147 | 0.078 | 0.124 | 0.065 | 1.901 | 0.065 | 0.021 |
|  | Interaction | 4.127 | 1.504 | 2.744 | 0.009 | 0.007 | 3.572 | 1.028 | 3.474 | 0.001 | 0.001 |
| Change in Between-ness | Intercept | -0.535 | 0.551 | -0.971 | 0.333 | 0.315 | -0.872 | 0.575 | -1.517 | 0.131 | 0.111 |
|  | Prop. affect | 1.49 | 1.619 | 0.92 | 0.363 | 0.399 | 1.704 | 1.655 | 1.029 | 0.31 | 0.328 |
|  | Treatment | 1.317 | 0.709 | 1.857 | 0.072 | 0.044 | 1.189 | 0.616 | 1.929 | 0.062 | 0.052 |
|  | Week | 0.124 | 0.173 | 0.718 | 0.477 | 0.427 | 0.277 | 0.172 | 1.608 | 0.117 | 0.073 |
|  | Interaction | -6.683 | 3.777 | -1.769 | 0.085 | 0.064 | -5.825 | 2.867 | -2.032 | 0.05 | 0.041 |
| Change in Average Strength | Intercept | 0.015 | 0.013 | 1.151 | 0.251 | 0.226 | 0.026 | 0.015 | 1.738 | 0.084 | 0.065 |
|  | Prop. affect | -0.07 | 0.037 | -1.871 | 0.07 | 0.028 | -0.144 | 0.044 | -3.309 | 0.002 | 0.001 |
|  | Treatment | 0.002 | 0.016 | 0.11 | 0.913 | 0.909 | -0.004 | 0.016 | -0.28 | 0.781 | 0.789 |
|  | Week | -0.008 | 0.004 | -2.053 | 0.047 | 0.035 | -0.007 | 0.004 | -1.495 | 0.144 | 0.122 |
|  | Interaction | 0.151 | 0.084 | 1.791 | 0.082 | 0.073 | 0.208 | 0.071 | 2.946 | 0.006 | 0.002 |

**Table S2.** Results of full models corresponding to Figure 3 (main text) considering how removed birds’ dyadic pre-removal social association predict their (a) probability of association upon reintroduction and (b) association strength upon reintroduction. For both models, GLMMs with binomial error structure and logit-link function were used, and included individual identity as random effects and assessed the effect of the fixed effects of (i) ‘Pre-removal association’ of the removed bird to the non-removed bird (see methods), (ii) ‘Distance’ between the removed bird’s initial capture site to their location following reintroduction, (iii) ‘Time until resighting’ between their reintroduction and their first observation (iv) the week which the bird was removed. The coefficient, standard error, *z* value and the standard p value are provided, along with the p value calculated from the randomisations (P_rand_). In (b), the association strength upon reintroduction was modeled as the number of flocks the removed bird and non-removed bird co-occurred in (‘successes’) in relation to the number of flocks the removed bird and non-removed bird did not co-occurred in (‘fails’) as a binomial equivalent of the Simple Ratio Index (see methods).

|  |  | Coeff. | SE | *z* value | P | P_rand_ |
| --- | --- | --- | --- | --- | --- | --- |
| a) Probability of association upon reintroduction | Intercept | 9.426 | 2.877 | 3.276 | 0.001 | 0.001 |
|  | Pre-removal Assoc. | 8.379 | 2.134 | 3.926 | 0.001 | 0.001 |
|  | Distance | -0.12 | 0.041 | -2.931 | 0.003 | 0.001 |
|  | Time until resighting | -1.624 | 0.741 | -2.191 | 0.028 | 0.002 |
|  | Week | -0.957 | 0.289 | -3.304 | 0.001 | 0.001 |
| b) Association strength upon reintroduction | Intercept | -4.261 | 1.049 | -4.064 | 0.001 | 0.074 |
|  | Pre-removal Assoc. | 3.777 | 0.436 | 8.659 | 0.001 | 0.014 |
|  | Distance moved | -0.041 | 0.02 | -2.096 | 0.036 | 0.544 |
|  | Time until resighting | 0.803 | 0.376 | 2.134 | 0.033 | 0.128 |
|  | Week | 0.054 | 0.100 | 0.539 | 0.59 | 0.482 |
